# Video capsule endoscopy in overt and occult obscure gastrointestinal bleeding: Insights from a single‐center, observational study in Japan

**DOI:** 10.1002/deo2.354

**Published:** 2024-04-01

**Authors:** Anna Tojo, Tomohisa Sujino, Yukie Hayashi, Kenji J L Limpias Kamiya, Moe Sato, Sakurai Hinako, Yusuke Yoshimatsu, Satoshi Kinoshita, Hiroki Kiyohara, Yohei Mikami, Kaoru Takabayashi, Motohiko Kato, Haruhiko Ogata, Takanori Kanai, Naoki Hosoe

**Affiliations:** ^1^ Division of Gastroenterology and Hepatology Department of Internal Medicine School of Medicine Keio University Tokyo Japan; ^2^ Center for Diagnostic and Therapeutic Endoscopy School of Medicine Keio University Tokyo Japan; ^3^ Department of Gastroenterology Saitama Medical Center Saitama Japan

**Keywords:** double‐balloon enteroscopy, obscure gastrointestinal bleeding (OGIB), overt and occult OGIB, single‐balloon enteroscopy, video capsule endoscopy

## Abstract

**Objective:**

This study aimed to evaluate the use of video capsule endoscopy (VCE) in patients with obscure gastrointestinal bleeding (OGIB), compare cases of overt and occult OGIB, assess the rates of balloon‐assisted enteroscopy (BAE) interventions and rebleeding, and identify predictive markers of positive VCE findings.

**Methods:**

Medical records of 430 patients who underwent VCE for OGIB between 2004 and 2022 were analyzed. Occult OGIB was defined as IDA or positive fecal occult blood, whereas overt OGIB was defined as clinically imperceptible bleeding. We retrospectively analyzed demographics, VCE findings based on Saurin classification (P0, P1, and P2), outcome of BAE interventions, and rebleeding rates.

**Results:**

A total of 253 patients with overt OGIB and 177 with occult OGIB were included. P1 findings were predominant in both groups, with a similar distribution. The percentage of patients receiving conservative therapy was higher in P1 than in P2 for both overt and occult OGIB. BAE was more frequently performed in P2 than in P1 VCE (83.0% vs. 35.3% in overt OGIB, 84.4% vs. 24.4% in occult OGIB). The percentage of positive findings and intervention in total BAE performed patients were comparable in P1 and P2 of overt OGIB, whereas these percentages in P2 were more than P1 of occult OGIB.

**Conclusion:**

VCE effectively identified OGIB lesions requiring intervention, particularly occult OGIB lesions, potentially reducing unnecessary BAE. Rebleeding rates varied according to the VCE findings, emphasizing the importance of follow‐up in high‐risk patients.

## INTRODUCTION

Obscure gastrointestinal bleeding (OGIB) is a diagnostic challenge when it occurs in the small intestine. OGIB is divided into two types: overt OGIB, which represents clinically obvious bleeding, and occult OGIB, which manifests as positive fecal blood test results or iron‐deficiency anemia (IDA).^1^ Balloon‐assisted enteroscopy (BAE) and video capsule endoscopy (VCE) are used to survey the gastrointestinal tract. However, the selection of the most suitable method for identifying bleeding in the small intestine remains debatable. VCE is widely used as a noninvasive process,^2^, ^3^ while BAE allows for therapeutic or diagnostic interventions. Most guidelines^1^, ^4^, ^5^ recommend the use of VCE as the first‐line modality for mid‐GI bleeding, although the detection rate of the source of OGIB is comparable between VCE and BAE.^6^ In a meta‐analysis, the diagnostic rate of BAE after VCE was higher than that of BAE alone.^6^, ^7^ In addition, the findings of VCE provide an adequate basis for approaching BAE.^8^

Ongoing overt OGIB is self‐limiting but is associated with a risk of mortality after recurrence.^7^, ^9^, ^10^ Currently, the European Society of Gastrointestinal Endoscopy recommends performing VCE and BAE within 14 days of overt OGIB.^4^, ^11^ In addition, other groups have recommended BAE within 72 h following overt OGIB, albeit with limited evidence.^12^, ^13^ Several meta‐analyses related to BAE and VCE for OGIB have shown conflicting results regarding the best medical approach, especially regarding the time from bleeding to examination, best device, and clinical outcomes of examination and therapeutic intervention for OGIB.^11^, ^14^ Several studies have examined BAE interventions in patients with overt OGIB. However, there are few reports on the findings of VCE in occult and overt OGIB or on the clinical course of BAE. Moreover, clinical outcomes of BAE application and intervention performance after VCE in patients with overt or occult OGIB remain unclear. Herein, we retrospectively evaluated the positive rates of intervention and BAE findings in patients with overt and occult OGIB, based on VCE findings.

## METHODS

### Study design

This retrospective observational study was approved by the ethics committee of Keio University Hospital (ID 20160259). Data were collected from medical charts, and details of endoscopy findings were obtained using an Endoscopy Reporting System (Solemio ENDO; Olympus) and capsule workstation equipped with dedicated reading software on each platform (PillCam, Medtronic and Endocapsule; Olympus). Patients who underwent VCE at Keio University Hospital between August 2004 and May 2022 were included in this study.

### Definition of occult and overt OGIB

OGIB was divided into occult and overt OGIB, occult OGIB was defined as IDA and/or a positive fecal blood test. Overt OGIB was defined as recurrent or persistent clinically perceptible bleeding despite negative initial endoscopy (esophagogastroduodenoscopy and colonoscopy) and radiological tests (small bowel follow‐through or enterohemolysis).^1^, ^15^ Only the first VCE result was included in the analysis in cases in which more than one examination was performed on the same individual.

### Data collection

Data collected included age, sex, type of VCE used, VCE completion rate, symptom/s, days from last bleeding to VCE, follow‐up duration after initial VCE (days), comorbidities such as diabetes mellitus, hemodialysis, laboratory data (hemoglobin (Hb, g/dL)), prescribed drugs (non‐steroidal anti‐inflammatory drugs [NSAIDs]), antiplatelet drugs, direct oral anticoagulants, prednisolone), and endoscopic information (VCE findings, whether BAE was performed after VCE administration or therapeutic intervention for BAE).

### Capsule endoscopy

The capsule types used were PillCam SB, SB2, and SB3 (Medtronic), and Endocapsule EC1 and EC10 (Olympus).

### Balloon‐assisted enteroscopy

Two types of BAE were used at our hospital: double‐balloon endoscopy (DBE; model: EN‐450T5/W, EN‐580T; Fuji Film) and single‐balloon endoscopy (SBE; model: SIF‐Q260, SIF‐H190; Olympus).

### Lesion detection by VCE

VCE findings are classified into three categories, as reported by Saurin et al.^16^: P0, lesions with no potential for bleeding; P1, lesions with uncertain hemorrhagic potential; and P2, lesions considered to have a high potential for bleeding. P2 lesions were defined as those with positive VCE results. Capsule reading and judgment were made by two VCE expert physicians with more than 200 cases of experience in VCE reading. In cases of disagreement between them, mutual discussions were held and consensus was reached.

### Primary and secondary endpoints

The primary endpoint was the rate of positive findings and intervention with BAE after VCE for overt and occult OGIB to evaluate the clinical impact of VCE. We determined lesions, such as erosions, vascular lesions, polyps, tumors, and ulcers, which have the potential to bleed as positive findings. We also defined therapeutic interventions as endoscopic treatment with BAE, such as mucosal resection or clipping, surgery, and chemotherapy after BAE diagnosis.

The secondary endpoints were rebleeding rate after VCE for overt OGIB and a predictive marker of positive VCE findings to evaluate the clinical impact of VCE.

### Statistical analysis

Statistical analysis was performed using the Student's t‐test for normally distributed continuous variables, the Mann–Whitney U test for non‐normally distributed continuous variables, and the Chi‐square test for the positive detection rate by BAE for patients who underwent VCE. For overt and occult OGIB, case‐control analysis was performed using univariate analysis to identify all potential factors. After determining the relevant risk factors in the univariate analysis, they were entered into multivariate analysis using a logistic regression model. Statistical significance was set at *p* < 0.05. SPSS version 27 (IBM) was used for all the statistical analyses.

## RESULTS

### Patient enrollment in the study

**FIGURE 1:**
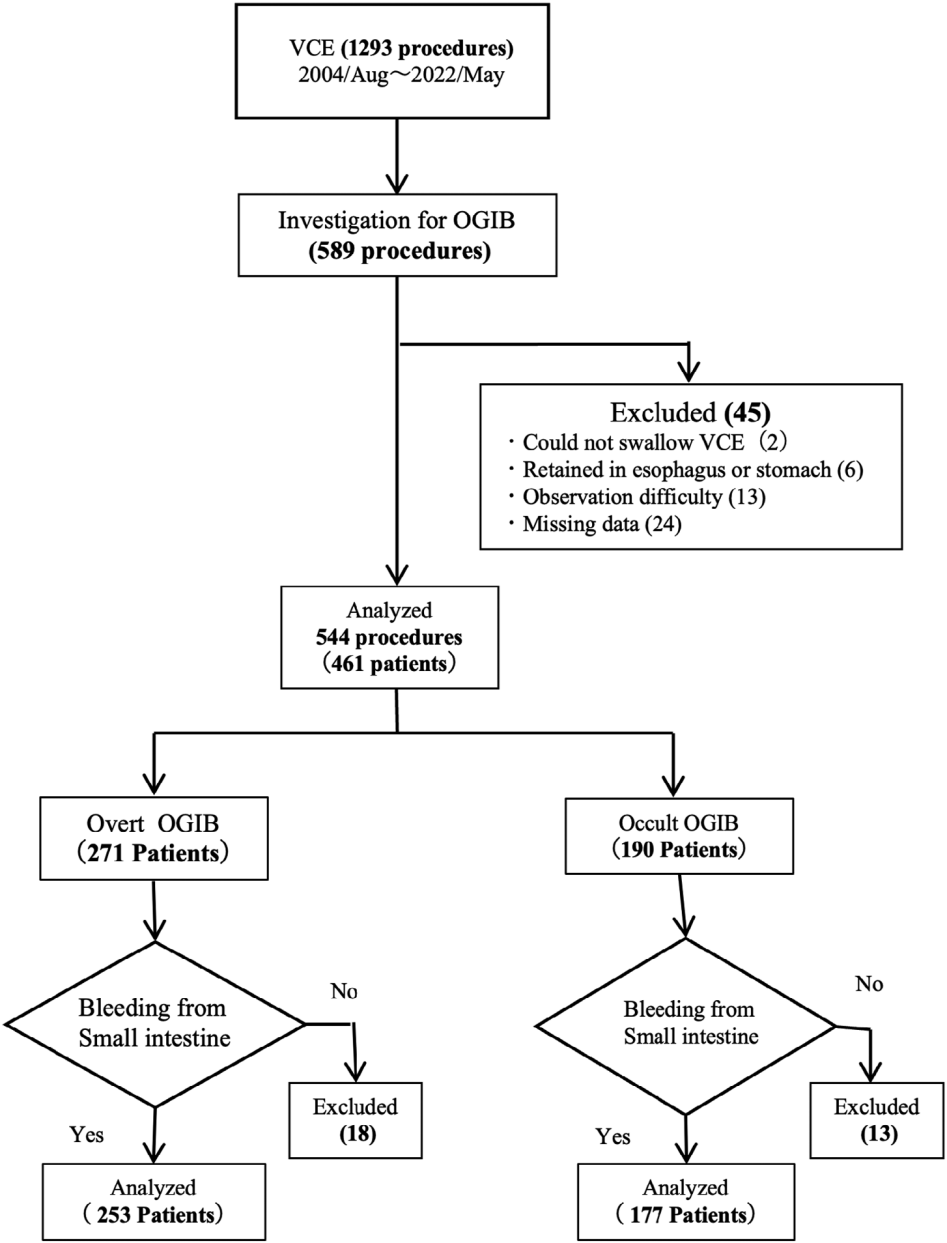
Flow diagram of enrolled patients. Flow diagram of patient enrollment VCE, video capsule endoscopy; OGIB, obscure gastrointestinal bleeding.

A total of 1293 patients underwent VCE at our hospital during the study period, of whom 589 were examined for OGIB. The final analysis included 253 and 177 patients with overt and occult OGIB, respectively (Figure 1).

### Clinical characteristics

The mean age was 63.34 ± 16.3 for overt OGIB and 67.46 ± 14.27 for occult OGIB, and the male‐to‐female ratio was 159/94 and 120/57, respectively; both results showed a male preponderance (Table 1). We did not observe any notable differences in the device selection. The overall small bowel observation rate was 88.1% for overt OGIB and 91.5% for occult OGIB. The clinical symptoms that triggered the VCE are presented in Table 1. The comorbidities and prescribed drugs are listed in Table 1. Blood laboratory findings confirmed Hb levels at the time of VCE, and both overt and occult OGIB had a mean value in the 9.9 g/dL range. Medications, medical history (DM, hemodialysis), and blood test results were analyzed, and there was no clear significant difference between the two groups.

**TABLE 1:** Patients’ clinical characteristics.

	Overt OGIB (*n* = 253)	Occult OGIB (*n* = 177)
Age (years), mean ± SD	63.34 ± 16.30	67.46 ± 14.27
Male/female	159/94	120/57
Type of VCE		
SB	49	30
SB2	42	22
SB3	89	47
EC1	35	40
EC10	38	38
VCE completion rate (%)	88.1 (223/253)	91.5 (162/177)
Symptom		
Hematochezia	69	−
Melena	181	−
Hematemesis	3	−
Iron‐deficiency anemia	−	163
Fecal occult blood test	−	14
Days from last bleeding to VCE (days), mean ± SD	22.06 ± 24.4	−
Follow‐up duration after initial VCE (days), mean ±SD	1127.1 ± 1337.30	1159.95 ± 1346.9
DM (+/‐) (%)	23/ 230 (9.1)	12/165 (6.8)
HD (+/‐) (%)	9/244 (3.6)	14/163 (7.9)
Blood test (at the time of VCE)		
Hb (g/dL), mean ± SD	9.978 ± 2.36	9.93 ± 2.17
Drug		
NSAIDs (+/‐) (%)	35/218 (13.8)	29/148 (16.4)
Antiplatelet drug (+/‐) (%)	56/197 (22.1)	41/136 (23.2)
DOAC (+/‐) (%)	10/243 (4.0)	14/163 (7.9)
Prednisolone (+/‐) (%)	24/229 (9.5)	14/163 (7.9)

Abbreviations: DM, diabetes mellitus; DOAC, direct oral anticoagulants; HD, hemodialysis; NSAIDs, nonsteroidal anti‐inflammatory drugs; OGIB, obscure gastrointestinal bleeding; VCE, video capsule endoscopy.

### Characteristics of VCE findings and side effects of the VCE performance

We then analyzed the types of VCE findings in overt and occult OGIB. Overall, the distribution of VCE findings in the None‐P0, P1, and P2 groups was similar between overt and occult OGIB groups (Table 2). Capsule retention occurred in three of 430 cases (0.7%), all of which were removed endoscopically.

**TABLE 2:** Video capsule endoscopy findings.

VCE findings	None or P0	P1	P2	Total
Overt (*n* = 253)				
Angiodysplasia	0	32	10	42
Bleeding	0	0	19	19
Cavernous lymphagioma	0	0	1	1
Diverticulum	1	0	0	1
Erosion	0	47	7	54
Meckel's diverticulum	0	0	3	3
Polyp	0	1	3	4
Redness	0	22	1	23
SMT	0	2	3	5
Tumor	0	0	5	5
Ulcer	0	4	11	15
Ulcer scar	0	1	2	3
Others	12	4	0	16
None	62	0	0	62
Total (%)	75 (29.6)	113 (44.6)	65 (25.7)	253 (100)
Occult (*n* = 177)				
Angiodysplasia	0	20	13	33
Bleeding	0	0	11	11
Cavernous lymphagioma	0	0	0	0
Diverticulum	0	0	0	0
Erosion	0	38	8	46
Meckel's diverticulum	0	0	1	1
Polyp	0	2	6	8
Redness	0	13	0	13
SMT	0	2	2	4
Tumor	0	0	4	4
Ulcer	0	0	5	5
Ulcer scar	0	1	0	1
Others	8	2	1	11
None	40	0	0	40
Total (%)	48 (27.1)	78 (44.1)	51 (28.8)	177 (100)

Abbreviations: SMT, submucosal tumor; VCE, video capsule endoscopy.

### BAE Interventions after VCE

For each category of VCE findings (None or P0, P1, or P2), we analyzed whether BAE was subsequently performed. As we did not perform BAE for patients with P0 by VCE, we analyzed the percentage of P1/P2 cases that were eligible for intervention (Table 3). In overt OGIB, 38.9% (45/113) of P1 VCE and 18.5% (12/65) of P2 VCE were treated with conservative intervention, such as discontinuation of anticoagulants or NSAIDs, resulting in the remaining cases being eligible for the intervention (*p* = 0.004). After excluding conservative cases, 35.3% (24/68) of P1 VCE patients and 83.0% (44/53) of P2 VCE patients underwent BAE (*p* < 0.001). Nine cases of P2 VCE in overt OGIB did not undergo intervention because of the lack of patient concentration or poor general condition.

**TABLE 3:** Progress after video capsule endoscopy findings.

Overt OGIB			
Progress	P1 (n)	P2 (n)	*p*‐value
Total number of VCE	113	65	
Conservative intervention (not with BAE)% (n/n)	38.9 (45/113)	18.5 (12/65)	0.004
Patients excluded conservative intervention (*n*)	68	53	
BAE performed% (n/n)	35.3 (24/68)	83.0 (44/53)	<0.001
BAE not performed (*n*)	44	9	
The lack of patient's concern (*n*)	0	7	
Poor general condition (*n*)	0	2	

Occult OGIB			
Progress	P1(n)	P2(n)	
Patients number with VCE	78	51	
Conservative intervention (not with BAE)% (n/n)	42.3 (33/78)	15.7 (8/51)	0.002
Patients excluded conservative intervention (*n*)	45	43	
BAE performed% (n/n)	24.4 (11/45)	84.4 (38/43)	<0.001
BAE not performed (*n*)	34	5	
The lack of patient's concern (*n*)	0	4	
Poor general condition (*n*)	0	1	

Abbreviations: BAE, balloon‐assisted endoscopy; VCE, video capsule endoscopy.

In occult OGIB, 42.3% (33/78) of P1 VCE and 15.8% (8/51) of P2 VCE were treated with conservative intervention, resulting in the remaining cases being eligible for the intervention (*p* = 0.002). After excluding conservative cases, 24.4% (11/45) of P1 VCE patients and 84.4% (38/43) of P2 VCE patients underwent BAE (*p* < 0.001). Five cases of P2 VCE in occult OGIB did not undergo intervention because of the lack of patient concentration or poor general condition.

Seventeen patients (14.5%, 17/117) underwent DBE and 100 patients (85.5%, 100/117) underwent SBE. As only a small number of patients underwent DBE, we did not distinguish between the cases performed by SBE and those performed by DBE (BAE cases).

We selected the root of BAE based on VCE. Oral BAE was selected for 41.2% (28/68) of the patients with overt and 40.8% (20/49) of the patients with occult OGIB (Table 4). We also selected trans‐anal BAE in 48.5% (33/68) of overt OGIB patients and 57.1% (28/49) of occult OGIB patients. Moreover, 10.3% (7/68) of patients with overt and 2% (1/49) of patients with occult OGIB underwent both transoral and trans‐anal BAE.

**TABLE 4:** The root of balloon‐assisted endoscopy (BAE) based on video capsule endoscopy (VCE).

Overt OGIB			
Root of BAE	P1	P2	Total
Oral BAE% (n/n)	33.3 (8/24)	45.5 (20/44)	41.2 (28/68)
Trans‐anal BAE% (n/n)	58.3 (14/24)	43.2 (19/44)	48.5 (33/68)
Both (oral and trans‐anal)% (n/n)	8.3 (2/24)	11.4 (5/44)	10.3 (7/68)

Occult OGIB			
Root of BAE	P1	P2	Total
Oral BAE% (n/n)	45.5 (5/11)	39.5 (15/38)	40.8 (20/49)
Trans‐anal BAE% (n/n)	58.5 (6/11)	57.9 (22/38)	57.1 (28/49)
Both (oral and trans‐anal)% (n/n)	0 (0/11)	2.6 (1/38)	2.0 (1/49)

Abbreviations: BAE, balloon‐assisted endoscopy; VCE, video capsule endoscopy.

### Primary endpoint for overt OGIB and occult OGIB

We analyzed the percentage of positive findings for BAE and the rate of intervention in patients who underwent BAE after VCE for overt and occult OGIB. The percentage of positive BAE findings was not significantly different between P1 VCE and P2 VCE in overt OGIB (75%, 18/24 and 84.0%, 37/44, respectively); however, in occult OGIB, the percentage of positive findings of P2 VCE (94.7%, 36/38) was higher than that of P1 VCE (72.7%, 8/11).

The number of patients treated as interventions by BAE is shown in Table 5. Seven patients (29.1%) in the P1 VCE of overt OGIB were treated with intervention. Of these, 85.7% had benign vascular lesions. Nineteen patients (43.1%) in the P2 VCE of overt OGIB were treated with interventions. Of these, 68.4% were benign vascular lesions and the rest were polyps, malignant tumors, and Meckel's diverticulum. The rates of intervention in P1 and P2 of the overt OBIG were not significantly different. On the other hand, in occult OGIB, only one patient in P1 VCE underwent BAE intervention and 50% of P2 VCE patients underwent intervention. Indeed, 26.3% (5/19) of the patients had polyps, and 21.1% (4/19) had malignant tumors. The rate of intervention in P2 VCE was higher than that in P1 VCE for occult OGIB.

**TABLE 5:** Balloon‐assisted endoscopy performed after video capsule endoscopy.

Overt OGIB	P1 (*n* = 24)	P2 (*n* = 44)	*p*‐value
Positive findings in BAE% (n/n)	75 (18/24)	84.0 (37/44)	0.3623
Intervention in BAE% (n/n)	29.1 (7/24)	43.1 (19/44)	0.2557
The reason for the intervention			
Benign vascular lesion% (n/n)	85.7 (6/7)	68.4 (13/19)	
Polyp% (n/n)	14.3 (1/7)	10.5 (2/19)	
Malignant tumor% (n/n)	0 (0/0)	10.5 (2/19)	
Meckel's diverticulum% (n/n)	0 (0/0)	5.3 (1/19)	
Anastomotic ulcer% (n/n)	0 (0/0)	5.3 (1/19)	

Occult OGIB	P1 (*n* = 11)	P2 (*n* = 38)	*p*‐Value
Positive findings in BAE% (n/n)	72.7 (8/11)	94.7 (36/38)	0.0337
Intervention in BAE% (n/n)	9.09 (1/11)	50.0 (19/38)	0.0102
The reason for the intervention			
Benign vascular lesion% (n/n)	100 (1/1)	52.6 (10/19)	
Polyp% (n/n)	0 (0/1)	26.3 (5/19)	
Malignant tumor% (n/n)	0 (0/1)	21.1 (4/19)	
Meckel's diverticulum% (n/n)	0 (0/1)	0 (0/19)	
Anastomotic ulcer% (n/n)	0 (0/1)	0 (0/19)	

Abbreviations: BAE, balloon‐assisted endoscopy; VCE, video capsule endoscopy.

### Secondary endpoint for overt OGIB

Of the 253 cases in the overt OGIB group, only 188 were able to undergo a follow‐up period of at least 60 days after VCE was evaluated for rebleeding after the examination, according to the classification of VCE findings (Table 6). Of 188 patients, 60 were eligible for none or P0, 82 for P1, and 46 for P2. Rebleeding occurred in five, 11, and 10 patients, respectively. The negative predictive value of None or P0 for overt OGIB (percentage of patients who did not bleed again during the follow‐up period) was 91.6% (55/60). However, Rebleeding in occult OGIB was not analyzed in this study.

**TABLE 6:** Rebleeding after video capsule endoscopy (VCE).

		Overt (*n* = 188)		
		None or P0 (*n* = 60)	P1 (*n* = 82)	P2 (*n* = 46)
Rebleeding	(+)	5	11	10
	(−)	55	71	36

### Predictors of the P2 findings in VCE

To investigate the predictive factors for P2 findings when VCE was performed in both overt and occult OGIB cases, we divided the cases with None/P0/P1 (non‐P2) or P2 in VCE (Table 7). In overt OGIB, Hb level (g/dL), days from the last bleeding episode to VCE, and use of low‐dose aspirin were significantly different between patients with non‐P2 and P2 by univariate analysis. Hb levels and the use of low‐dose aspirin were significant positive markers by multivariate analysis. In occult OGIB, hemodialysis was an independent predictor between the patients with non‐P2 and P2 by univariate and multivariate analysis.

**TABLE 7:** Predictors of "P2" in overt and occult obscure gastrointestinal bleeding (OGIB).

	Univariate			Multivariate	
	NONE, P0, and P1	P2	*p*‐value	OR (95% CI)	*p*‐value
Overt					
Number	188	65			
Factors					
Male/female	122/66	37/28	0.252		
Age (years), mean ± SD	63.3 ± 16.6	63.4 ± 15.5	0.985		
Hb (g/dL), mean ± SD	10.3 ± 2.45	9.17 ± 1.91	0.001	0.822 (0.714–0.945)	0.006
Days from last bleeding to VCE (days), mean ± SD	24.0 ± 25.7	16.89 ± 19.7	0.044	0.988 (0.973–1.003)	0.115
Use of two or more antithrombotic drugs (+/‐)	28/160	4/61	0.068		
Use of an antiplatelet drug (+/‐)	44/144	12/53	0.408		
Low‐dose aspirin (+/‐)	40/148	6/59	0.03	2.847 (1.127–7.194)	0.027
Clopidogrel (+/‐)	14/174	8/57	0.305		
Use of DOAC (+/‐)	7/181	3/62	0.72		
Apixaban (+/‐)	1/187	0/65	1		
Rivaroxaban (+/‐)	1/187	2/63	0.163		
Edoxaban (+/‐)	5/183	1/64	1		
Use of warfarin (+/‐)	24/164	7/58	0.672		
Use of prednisolone (+/‐)	18/170	6/59	0.935		
Use of NSAIDs (+/‐)	25/163	10/55	0.674		
Hemodialysis (+/‐)	6/182	3/62	0.698		
Medical history of DM (+/‐)	19/169	4/61	0.456		
Occult					
Number	126	51			
Factors					
Male/female	86/40	34/17	0.86		
Age (years), mean ± SD	66.17 ± 15.1	70.65 ± 11.5	0.059	1.025 (0.998–1.052)	0.068
Hb (g/dL), mean ± SD	9.71 ± 2.24	10.0 ± 2.15	0.394		
Use of two or more antithrombotic drugs (+/‐)	18/108	7/44	0.923		
Use of an antiplatelet drug (+/‐)	31/95	10/41	0.476		
Low‐dose aspirin (+/‐)	27/99	7/44	0.239		
Clopidogrel (+/‐)	6/120	5/46	0.3		
Use of DOAC (+/‐)	10/116	4/47	1		
Apixaban (+/‐)	4/122	1/50	1		
Rivaroxaban (+/‐)	2/124	0/51	1		
Edoxaban (+/‐)	3/123	3/48	0.357		
Use of warfarin (+/‐)	13/113	8/43	0.316		
Use of prednisolone (+/‐)	11/115	3/48	0.76		
Use of NSAIDs (+/‐)	20/106	9/42	0.773		
Hemodialysis (+/‐)	6/120	8/43	0.027	3.665 (1.195–11.247)	0.023
Medical history of DM (+/‐)	7/119	5/46	0.331		

Abbreviations: BAE, balloon‐assisted endoscopy; DM, diabetes mellitus; DOAC, direct oral anticoagulants; Hb, hemoglobin; NSAIDS, non‐steroidal anti‐inflammatory drugs; OGIB, obscure gastrointestinal bleeding; VCE, video capsule endoscopy.

## DISCUSSION

We categorized BAE interventions and clinical outcomes based on VCE findings for both overt and occult OGIB. The prevalence of P1 and P2 lesions on VCE was similar between overt and occult OGIB. Furthermore, patients with P2 lesions in overt and occult conditions (Overt‐P2_VCE_ and Occult‐P2 _VCE_ groups, respectively) were more likely to undergo BAE than those with P1 lesions (Overt‐P1_VCE_ and Occult‐P1 _VCE_ groups, respectively). We did not observe a substantial difference in positive findings of BAE between overt and occult OGIB patients. Interestingly, the intervention rate of BAE in the Occult‐P2_VCE_ group was higher than that in the Occult‐P1_VCE_ group, whereas the intervention rate of BAE was comparable between the Overt‐P1_VCE_ group and the Overt‐P2_VCE_ group. Moreover, almost half of the patients in the Occult‐P2_VCE_ group expressed polyps and malignant tumors. This finding suggests the importance of P2 lesions in occult OGIB, as well as in both P1 and P2 lesions in overt OGIB for VCE.

Male preponderance was noted in overt OGIB cases, as previously reported,^17^ we found that male preponderance was also observed in occult OGIB cases. The distribution of VCE findings at P0, P1, and P2 in overt and occult OGIB was similar. While vascular lesions were more frequently detected in overt OGIB,^18^ small lesions, such as erosions and angiodysplasia, were prominent in the Overt‐P1_VCE_ and Occult‐P1_VCE_ groups in this study.

In occult OGIB, the precise date of the last bleeding event is unknown, and the timing of VCE is often later than in overt OGIB. Nevertheless, the finding of P2_VCE_ in 30% of occult OGIB cases is unexpected. These results suggest that VCE may be useful for identifying gross lesions that would result in P2_VCE_ findings, even in occult OGIB, regardless of whether anemia or fecal occult blood positivity was noted.

BAE should be performed if there is a finding of VCE, according to the Japanese guidelines.^19^

In real‐world data, conservative therapy was selected because of capsule findings, especially in P1 findings in both overt and occult OGIB. We tend to choose BAE in patients with P2 findings in both overt and occult OGIB. Nevertheless, around one to the third or fourth patients who expressed P1_VCE_ for both overt and occult OGIB underwent BAE. In addition, the percentage of positive BAE findings and intervention rate in BAE were comparable between the Overt‐P1_VCE_ and Overt‐P2_VCE_ groups, whereas the percentage of positive BAE findings and intervention rate in BAE in the Occult‐P2_VCE_ group was higher than that in the Occult‐P1_VCE_ group.

Notably, this selection bias may have affected the high ratio of positive findings in the number of BAE procedures in our study. Indications for BAE and VCE for overt and occult OGIB may differ in clinical settings, especially in the real world. Indication for BAE in occult OGIB might differ from that in overt OGIB.

BAE might be reconsidered for the Occult‐P1_VCE_ group because the probability of therapeutic intervention is low, despite the high frequency of findings. Also, almost half of the patients in the Occult‐P2_VCE_ group had polyps and malignant tumors. These data suggest the effectiveness of VCE and BAE in identifying intestinal diseases in patients with the Occult‐P2_VCE_ group.

When overt OGIB is judged as P0_VCE_ or P1_VCE_ with a negative predictive value as a filter, a certain number of cases of rebleeding are possible. Further studies are needed to estimate the risk of rebleeding in P0_VCE_ and P1_VCE_ patients based on VCE and patient background, such as dialysis and liver cirrhosis.^20^ Herein, we observed 8–20% rebleeding 60 days after the intervention. As previous reports have indicated greater rebleeding in the first year after 5 years of follow‐up,^20^, ^21^ rebleeding rate was lower than that previously reported due to the shorter observation period.

Predictors significantly related to the detection of lesions or rebleeding include the elderly, use of antithrombotic drugs and NSAIDs, high prevalence of vascular lesions in dialysis patients, and scattered reports of VCE performed within a short time after bleeding.^18^, ^20^, ^22^, ^23^, ^24^ In our analysis, patients taking antiplatelet drugs were more likely to have P2_VCE_ findings. It is possible that there was no difference between antithrombotic drugs and DOACs due to the limited number of patients who received these drugs. The possibility that P2_VCE_ findings do not correlate with rebleeding cannot be ruled out and further studies are required.

The limitations of the current study include the fact that it was a single‐center, retrospective, observational study and not a multicenter study. Although the third‐generation capsule endoscope (PillCam SB3) is the most recent VCE type, there were significant differences in the actual diagnostic rates of SB3 and SB2.^25^ In addition, we did not distinguish between BAE types. Indeed, DBE can typically advance deeper into the small intestine than SBE.^26^, ^27^, ^28^ Nevertheless, the disease detection and treatment success rates were comparable between SBE and DBE.^27^, ^29^, ^30^, ^31^

## CONCLUSION

BAE intervention rate was analyzed based on VCE findings in overt and occult OGIB. VCE may be useful in determining BAE and interventions not only in overt but also in occult OGIB.

## CONFLICT OF INTEREST STATEMENT

Naoki Hosoe conducted the independent research supported by funding contributions from Covidien. Naoki Hosoe received a research grant from Olympus. Naoki Hosoe is an associate editor of DEN Open. The other authors declare no conflict of interest.
